# Passive Visible Light Detection of Humans

**DOI:** 10.3390/s20071902

**Published:** 2020-03-29

**Authors:** Kenneth Deprez, Sander Bastiaens, Luc Martens, Wout Joseph, David Plets

**Affiliations:** WAVES, Department of Information Technology (INTEC), Ghent University/imec, Technologiepark-Zwijnaarde 126, B-9052 Ghent, Belgium; sander.bastiaens@ugent.be (S.B.); luc1.martens@ugent.be (L.M.); wout.joseph@ugent.be (W.J.)

**Keywords:** visible light sensing (VLS), passive VLS, human pass-by detection

## Abstract

This paper experimentally investigates passive human visible light sensing (VLS). A passive VLS system is tested consisting of one light emitting diode (LED) and one photodiode-based receiver, both ceiling-mounted. There is no line of sight between the LED and the receiver, so only reflected light can be considered. The influence of a human is investigated based on the received signal strength (RSS) values of the reflections of ambient light at the photodiode. Depending on the situation, this influence can reach up to ±50%. The experimental results show the influence of three various clothing colors, four different walking directions and four different layouts. Based on the obtained results, a human pass-by detection system is proposed and tested. The system achieves a detection rate of 100% in a controlled environment for 21 experiments. For a realistic corridor experiment, the system keeps its detection rate of 100% for 19 experiments.

## 1. Introduction

Indoor positioning has become increasingly important in daily life, which is a logical development as people spend about 80%-90% of their time indoors [1]. Indoor positioning can be used for a manifold of applications in various environments. Indoor localization can be used to navigate in supermarkets or give location-based discounts [2]. In hospitals, it can be used to find a specific piece of equipment or to navigate visitors or staff. Localization can help first responders for emergency support and disaster management. In industry, it is utilized to track and navigate automatic guided vehicles or unmanned aerial vehicles in a warehouse [3].

Positioning is and has been a popular research topic. Global Positioning System (GPS) dominates outdoor localization, thanks to improvements in both hardware and software (https://gssc.esa.int/navipedia/index.php/GPS_Future_and_Evolutions). Moreover, GPS is free to use contributing to its dominance. However, GPS is unreliable in built-up areas or indoors as no line of sight (LOS) connection is available. In addition, GPS suffers from signal attenuation on through material propagation [4]. The limited positioning accuracy of several meters [5] is not sufficient for many indoor localization use cases. Consequently, other solutions have been proposed for indoor localization using i.a. acoustic sensors, infrared detectors, radar, camera, chemical sensors or signals originating from a device assumed to be in the possession of a person [6]. These solutions all suffer from one or more of the following drawbacks: insufficient accuracy (i.e., no cm order positioning), expensive in either use or installation, spectrum congestion, electromagnetic (EM) interference, privacy violations and insufficient coverage.

Based on the advances in LED lighting, a new solution has recently attracted attention. Visible light positioning (VLP) uses visible light by modulating the light output of illumination sources. There is an ongoing discussion among researchers on the sender/receiver structure and modulation algorithm to use but regardless of the hardware and the algorithm, VLP provides an answer to the previously listed drawbacks. As light can not leave a room (i.e., if the door is shut and the walls are opaque), the communication link between sender and receiver is inherently safe. Moreover, the identity of the receiver or the person in possession of the receiver can remain unknown, preventing privacy violations. Besides, visible light is safe to use in EM sensitive environments. As illumination is present in typical indoor environments, it requires virtually no additional infrastructure to create a VLP-enabled environment. Simulation results indicate centimeter level accuracy for typical system parameters [7], while practical experiments report an accuracy of 0.1 to 0.2 m [8,9].

Although VLP can solve many of the previously listed drawbacks, some challenges still need to be solved to achieve worldwide employment of VLP. The first challenge is multi-path reflections. The positioning accuracy is limited in corners due to these reflections [10]. Secondly, VLP systems need LOS connections to achieve accurate positioning accuracy. Lastly, tilt introduces a constant error that needs to be tackled. Tilt can occur at the receiver as well as at the emitter [11].

Recently, a variant on VLP is being investigated—Visible Light Sensing (VLS). VLP focuses on localization while VLS focuses on presence detection of certain objects, mostly humans. VLS will exploit the influence of the object on the reflection pattern within the environment. This influence is used for sensing and can be used to improve localization accuracy of VLP. Furthermore, a hybrid system with any local positioning technology can be made.

A smart lighting and heating system in a typical office environment can be implemented based on visible light human sensing. Inefficient lighting and heating lead to high energy consumption and unnecessary costs. By installing efficient lighting and advanced lighting controls, costs can be reduced with 30% to 50% [12]. Current sensing technologies consist mainly out of infrared (IR) sensors. The person is detected on entering and on movement. If the human becomes immobile, the IR sensor will no longer detect the human, so the lights will switch off. In a VLS system, the human is still detected, even when standing still, which is an improvement over IR-based sensing systems.

In this paper, a passive VLS system is tested. A passive VLS system consists of unmodulated light and a receiver that is fixed in the environment. Wang et al. [13] listed the challenges and opportunities of passive VLS. There is no control over the objects’ shape, which implies no one-size-fits-all solution. Receivers can only sense an object when it moves under the limited field-of-view (FOV) of the receivers. Hence, high-density employment of receivers will be required to provide fine-grained results. Nevertheless, research is performed to limit the number of receivers by focusing on smart deployment.

Light nodes, including both the LED light and the photodiodes, are proposed in Reference [14] to detect objects passively. The LED is modulated, so the source of incident light can be identified at the photodiode (PD) based on the received frequencies. Experiments detect whether a door is open or closed. Based on this work, Eyelight [15] is an extension that focuses on human movement and occupancy detection. Occupancy detection, using a machine learning approach, achieves an overall classification accuracy of 93.7% in a 45 m${}^{2}$ room.

A first full passive system is described in [16]. Battery-free radio-frequency identification (RFID) sensor tags equipped with photodiodes are embedded in the floor. The receivers detect a shadow caused by a human that passes. When a test subject walks slowly (0.32ms), there is a 100% detection rate. If the test subject walks faster (0.75ms), the detection rate drops to 93.3%. However, as the receivers are embedded in the floor, a large scale deployment is infeasible.

Another full passive system is found in Reference [17]. Ceilingsee uses a modified LED that functions both as receiver and as emitter. The focus of this paper is on occupancy detection in a meeting room. An accuracy above 90% can be obtained in an experimental environment for both static and dynamic scenarios, which indicates that VLS is able to perform human detection accurately.

To the best of the authors’ knowledge, this paper is the first reported work on the influence of a single human on the reflection pattern for visible light in a LED-PD ceiling-mounted environment. It offers the following contributions:

1. *First reported experimental results on the influence of a human on received signal strength:* the influence of clothing color and walking direction of a test subject on the received signal strength are investigated;
2. *Influence of receiver height on received signal strength:* experimental results for a room height of 3 and 4 m are reported for a single LED, single PD deployment;
3. *Experimental human detection system:* a human detection system is tested using various test subjects. A 100% detection rate is achieved in a controlled lab environment;
4. *Influence of separation between LED and PD is experimentally assessed:* different LED-PD separation distances are investigated (30 cm to 150 cm). The influence of the distance between LED-PD is significant.

The outline of this paper is as follows—Section 2 provides information about the lab environment and the testing methods used to gather the ΔRSS plots presented in Section 3. Section 4 investigates the possibility to design a robust human pass-by detection system. Section 5 discusses two important human sensing scenarios. Finally, future work and conclusions are summarized in Section 6.

## 2. Measurement Method and Setup

### 2.1. Default Lab Environment

**Figure 1:**
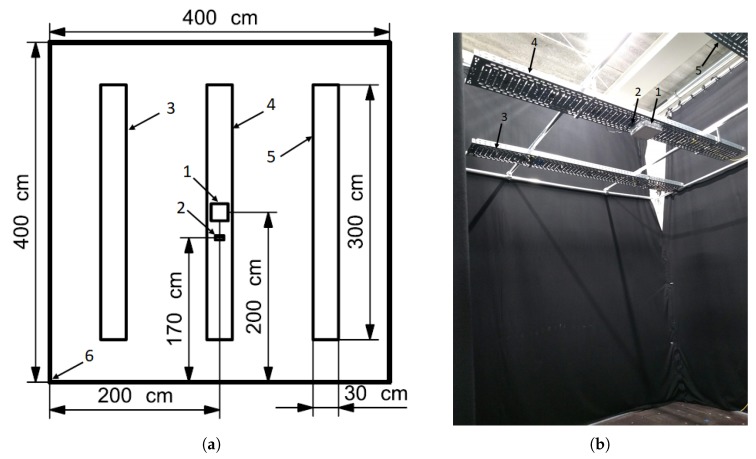
(**a**) The top view of the layout, (**b**) A picture of the lab with (1) the LED panel, (2) the optical receiver, (3) first rail, (4) second rail, (5) third rail, (6) the origin of the lab (location (0,0)).

Figure 1a shows the layout of the considered environment of 4 m by 4 m. The ceiling of the lab consists of three rails that are adjustable in height. Both the LED and the receiver are mounted on the ceiling. The ceiling in the default environment is placed at a height of 3 m. The walls of the lab are four black cloths mounted on the aluminum construction that carries the ceiling in order to avoid uncontrollable reflections. However, it is expected that the impact of reflections on the walls is limited as these already include at least two reflections. The floor in the lab consists of wooden panels. A picture of the lab is also included (Figure 1b).

A simple off-the-shelf 18W 7000K floodlight LED panel (20 × 20 cm) is used as light source (Tx). The center of the LED panel corresponds with the center of the room (200,200). The used optical receiver, a photodiode (PD), is the PDA100A2 from Thorlabs [18]. The center of the PD (Rx) is mounted at y = 170 cm, while the x location equals x = 200 cm. The gain of the PD is adjustable and is set to 1.5×106VA for the experiments.

When light falls on the active area of the PD, that is, the photosensitive area, a “weak” photocurrent will be generated. The PD will convert this photocurrent into a voltage using its internal transimpedance amplifier circuit. Changes in incident light will result in a changing output voltage. An Analog to Digital Converter (ADC) reads the output voltage. The used ADC is a USB-6212 from National Instruments (http://www.ni.com/pdf/manuals/375196d.pdf), which has a resolution of 16 bits and a timing resolution of 50 ns. The obtained output is sent to a computer (Dell Latitude E7240, Intel Core i7-4600U Processor CPU @ 2.10 GHz 2.70 GHz) where a MATLAB^®^ script processes and stores the data.

Both the LED and the photodiode (PD) are ceiling-mounted in these experiments, the influence of an object on the reflection patterns within the environment is the main subject of this paper. As stated before, the PD will convert the received incident light to an output voltage. The used metric for this paper is Received Signal Strength (RSS) and is expressed in millivolt (mV). Following the fact that both the PD and the LED are ceiling-mounted for an easier and more cost-effective real-life deployment, only Non Line Of Sight (NLOS) light, that is, reflections will reach the PD. This provides a challenge as the power of the incident light is low due to a long path length and absorption of light at the reflectors.

An *RSS value* is a single measurement during a specific time frame. As the sample rate of the PD is high (256×103sampless), an RSS value in this paper is the average of $256\times 10^{3}$ samples, that is, all mV values obtained during one second. An *RSS lapse* is defined as multiple subsequent RSS values set out on a time axis and contains temporal data about a specific location. An *RSS pattern* consists of multiple RSS values at different locations and contains spatial information. An RSS lapse is displayed in a graph, while an RSS pattern is presented in a contour plot.

### 2.2. DC Light

**Figure 2:**
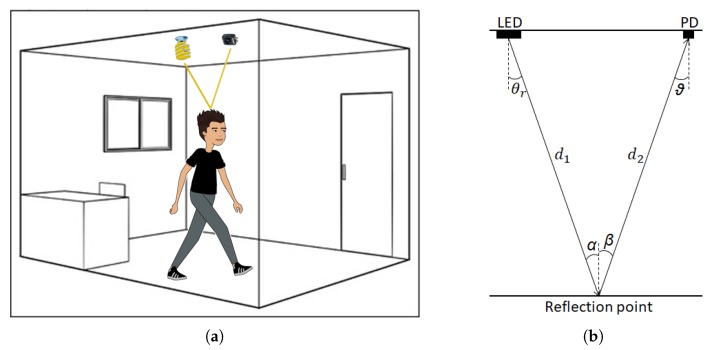
(**a**) One “perfect” light ray. One light and one PD are mounted at the ceiling. (**b**) A schematic diagram of Equation (2).

The only incident light in the PD is due to reflections. If a human is present and standing in the vicinity of the LED-PD combination, the human is expected to have a great influence on the RSS value. Figure 2a shows one “perfect” light ray, that is, a light ray that reflects straight to the PD is shown.

The received power at the PD is usually divided into a Line Of Sight (LOS) and a Non LOS (NLOS) component and can be expressed as in [19]:

(1) $$P_{r}=P_{t}H_{d}\left(0\right)+\int P_{t}H_{ref}\left(0\right),$$

where $H_{d}\left(0\right)$ and $H_{ref}\left(0\right)$ are the DC channel gain of the direct and reflected paths respectively and $P_{t}$ is the total optical power transmitted by the LED.

In most literature, however [7,20], the NLOS component is ignored as its contribution is negligible. Here, there is no LOS path between the receiver and the light, so only the NLOS component remains. The DC gain of the first reflection can be written as in Reference [21]:

(2) $$H_{ref}\left(0\right)=\left\{\begin{array}{cc} \frac{\left(m+1\right)}{2\pi d_{1}^{2}d_{2}^{2}}\rho A_{det}dA_{w}cos^{m}\left(\theta_{r}\right)cos\left(\alpha \right)cos\left(\beta \right)T_{s}\left(\vartheta \right)g\left(\vartheta \right)cos\left(\vartheta \right), & for\;0\leq \vartheta \leq |\vartheta_{FOV}|\\ 0,\;\;\;\;\;\;\;\;\;\;\;\;\;\;\;for\;\vartheta >|\vartheta_{FOV}| & , \end{array}\right.$$

where $d_{1}$ is the distance between transmitter and reflection point, $d_{2}$ is the distance between the reflection point and receiver, ρ is the reflectance coefficient, $dA_{w}$ is a small reflective area on the wall, $\theta_{r}$ is the angle of irradiance, ϑ is the angle of incidence, $T_{s}\left(\vartheta \right)$ is the optical filter gain, g(ϑ) is the optical concentrator gain and $\vartheta_{FOV}$ is the Field Of View (FOV) of the receiver, α is the angle of incidence from the transmitter and β is the angle of irradiance from the small reflective area. A schematic diagram is shown in Figure 2b.

The emitted power of the LED can be considered constant throughout the entire duration of the experiments. The $RSS_{empty}$ value is recorded in a humanless environment and acts as a base value. This base value contains the reflections on the walls, floor and static objects (e.g., desk, chair and closet) if present. Future measurements, where a human is present ($RSS_{person}$), are compared against this base value so a relative RSS value (ΔRSS) is obtained that only considers the reflections and shadows caused by a human. These relative RSS values can be negative if the reflected power decreases due to the presence of a human. The base value is measured before the start of every measurement. This results in:

(3) $$\Delta RSS=RSS_{person}-RSS_{empty}.$$

### 2.3. Experiment Description

For this paper, two different experiments are conducted—full room (Section 2.3.1) and human detection (Section 2.3.2) experiments. The former collects spatial data of the entire environment. Here, various scenarios are tested to investigate the influence on the reflection pattern. In the latter, temporal data is collected to perform human detection. The temporal data is then compared against a threshold to detect a test subject.

#### 2.3.1. Full Room Experiments

The full room experiments entailed the test subject advancing in steps of 10 cm from right to left (from x = 400 cm to x = 0 cm) according to the origin of the lab. As the lab is 4 m wide, 40 measurements are executed per y-value. After the test subject reached the end of a row, he/she moved to the next row, which is the current y-location +10 cm. By doing so, a grid on the floor is constructed with measurement points every 10 cm. The test subject followed several guiding lines inside the lab and was trained to take steps of 10 cm by several test runs. A marker was placed on the shoes of the test subject to indicate the step size. The average error that the test subject introduces by an incorrect step size is ±1.1 cm. This has been tested by letting the test subject perform ten steps and measure the error of those steps. The time between subsequent measurements is 2 s. The test subject will look down to focus on advancing correctly and then raise his head partially. Light reflecting on a slightly forward-tilted head still reflects on hair, allowing for similar results as when the subject would be looking forward. A slightly different reflection is expected when the subject would look upwards to the LED-PD, as the light then reflects on skin rather than on hair.

**Figure 3:**
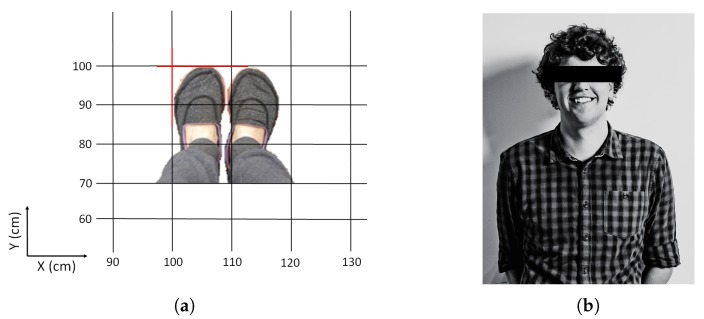
(**a**) Placement of the feet for location (100,100). (**b**) The test subject.

The guiding lines are used to indicate the location of the person. The left side of the shoe is placed against the guiding line as is the tip of the shoe (Figure 3a). Here, the tip of the shoe of the test person is placed at y = 100 cm, while the left side of the left foot is placed at x = 100 cm. However, the center of the body will not be at that exact position. Hence, a shift in the figures is expected according to the walking direction and the position of the center of the body.

##### Influence of the Environment

The lab environment can be altered. The ceiling of the lab is adjustable in height and is altered from 3 m, which was the default lab environment, to 4 m. As seen in (2), the distances between the transmitter and the reflection point ($d_{1}$) and between the reflection point and the receiver ($d_{2}$) influence the received power. The location of the light and the receiver can be changed as well. The distance along the y-axis between the LED and the PD is altered so that a more significant separation is obtained. By changing the LED-PD separation, the recorded RSS values will also change.

##### Influence of the Test Subject

As the goal of this research is to perform human sensing, an important parameter is the test subject. The appearance of this person influences the observed RSS values as blond hair is more reflective than black hair [22]. The hair type will entail a different RSS value as curly hair is expected to cause more diffuse reflections than straight hair [23]. A diffuse pattern is expected as the light is reflected between hair fibers before exiting again. The way one dresses also has an influence, as white fabric will reflect more light than black fabric. Some body parts are closer to the ceiling (e.g., shoulders, head), so their influence is greater as the path length is shorter as can be seen in (2).

For this paper, one test subject is considered as to compare the gathered results. The test person (Figure 3b) has curly brown hair and is 1 m 90 tall. The test subject has an average body shape and was not wearing any accessories.

#### 2.3.2. Human Detection Experiment

##### Lab Environment Experiment

For the human detection experiments, more dynamic tests were performed. The test subjects walked from right to left and had four seconds to reach the other side of the lab, which corresponds with a walking speed of 1ms. The y location of the PD was changed to y = 75 cm for these experiments as will be explained later. The test subjects also walked at a y location of y = 100 cm. The four seconds of data were divided into 40 subsets of 0.1 seconds each. The sample rate of the PD remained 256×103sampless. An average of each period (i.e., an average of $256\times 10^{2}$ samples) was saved and set out in the graph. The test subjects were chosen at random so no restrictions were made regarding appearance, accessories or clothing. Nineteen unique test subjects tested the human detection system. In total, 21 tests were performed. Two test subjects performed two tests using two different colored t-shirts.

##### Realistic Corridor Experiment

These tests were repeated in a real-life corridor. Again, 19 test subjects were selected at random. The sample rate and way of testing remained unchanged. The photodiode was mounted right next to a light fixture to best mimic an integrated lighting/sensing LED infrastructure. The tests were performed by day under varying ambient light conditions. As the system continuously measures RSS values, a time interval was created as soon as a person is detected. A sliding window determining the average RSS value in the most recent 10s-window in which the variation on the RSS was below 5% was considered as $RSS_{empty}$ in Equation (3). This ensures correct $RSS_{empty}$ levels to subsequently determine ΔRSS values.

## 3. Results

### 3.1. RSS Drop

The influence of different configurations on the reflection pattern within the room will be reported. Within every ΔRSS pattern (from (3)), an RSS drop, that is, a decrease in the RSS values will be observed. As a person is standing under and between the PD-LED combination, the reflections will scatter on the human and block the first order reflections on the floor. First order reflections are reflections that have reflected only once. These first order reflections lead to a higher received power and a higher influence on the ΔRSS pattern. However, the human will also introduce new first order reflections. A human shape is more rounded than a floor and therefore introduces a more diffuse pattern. The logical result of the presence of a human is thus that the ΔRSS value will reach a minimum, that is, a drop.

**Figure 4:**
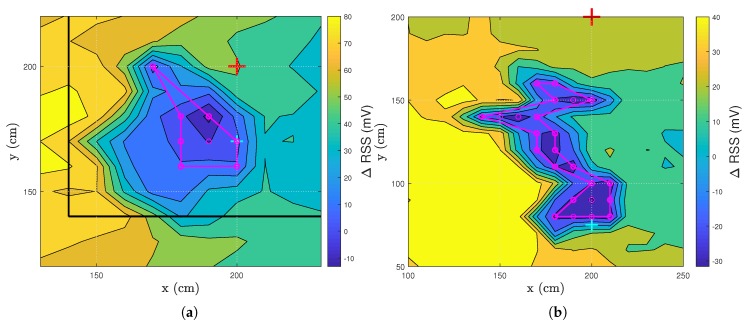
Detailed result of the ΔRSS drop of later mentioned results. (**a**) Layout 1, (**b**) layout 3. The red cross represents the LED, while the cyan cross denotes the PD. The black line represents the extent of the occurrence area.

**Figure 5:**
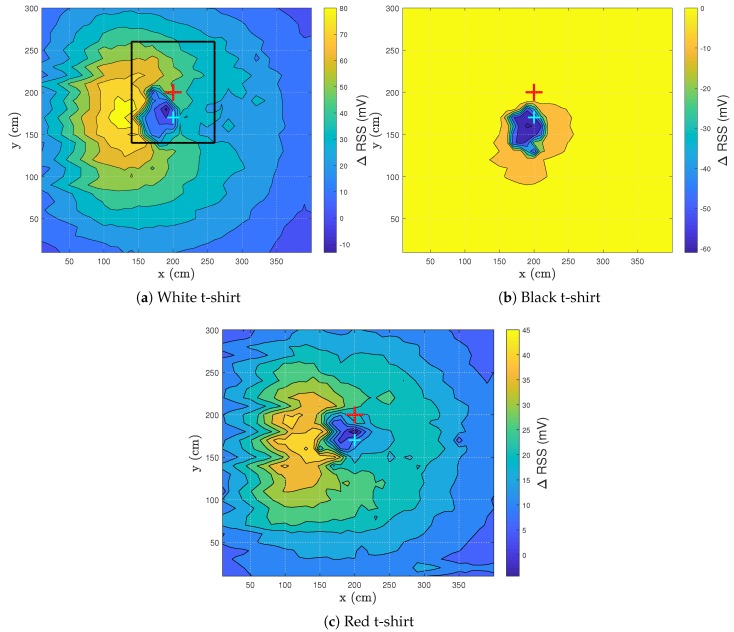
Visualization of the ΔRSS distribution for the test subject with a room height of 3 m wearing different colored t-shirts. The red cross represents the LED, while the cyan cross denotes the PD. The black square shows the occurrence area of the ΔRSS drop.

To objectify this RSS drop, certain assumptions are made. As stated before, the drop occurs in the vicinity of the LED-PD combination. Hence, the extent of the occurrence area of the ΔRSS drop is limited and is determined by the separation between the LED and the PD. Regarding the position of the LED as the center, the area spans two times the distance between the LED and the PD, creating a square. The occurrence area can thus be summarized as follows: $y_{LED}-2\;\cdot \;|y_{LED}-y_{PD}|<x,y<y_{LED}+2\;\cdot \;\left|y_{LED}-y_{PD}\right|$. $y_{LED}$ is the y location of the LED and $y_{PD}$ is the y location of the PD. In the default layout of the lab, the numerical values of the ranges are thus: 140cm<x,y<260cm with $y_{LED}$ = 200 cm and $y_{PD}$ = 170 cm. The extent of the occurrence area is shown by the black square in Figure 4a. The full extent can be seen in Figure 5a.

To find all locations that correspond to the RSS drop in the occurrence area, the RSS value has to be below a certain threshold. The threshold is calculated based on the difference between the maximum ($\Delta RSS_{max}$) and minimum ($\Delta RSS_{min}$) RSS values. For an RSS value to be included in the drop, the value has to be lower than 25% of all the RSS values, which is an empirically chosen value based on the area of the lab environment and the field of view (FOV) of the receiver. This is summarized in following equation defining the threshold for an RSS drop range

(4) $$\begin{array}{c} \begin{array}{c} Threshold=0.25\;\cdot \;\left(\Delta RSS_{max}-\Delta RSS_{min}\right)+\Delta RSS_{min}. \end{array} \end{array}$$

This is a labor-intensive way to determine a threshold as it requires a full room measurement. Further research is needed to determine an accurate threshold based on fewer measurements. When historical data is available (from similar environments), machine learning can be used to predict thresholds. Furthermore, researchers are focusing on accurate modeling of visible light propagation. If the source, ambient light and first order reflections can be characterized accurately, thresholds could be predicted based on simulated data. The extent of the occurrence area and the place where the ΔRSS drop occurs in regard to the LED-PD will remain valid in other environments.

Based on this drop, a manifold of applications can be created. In this research, two are considered. The first is to create a human pass-by detection system. As the movement of a human introduces the RSS drop, he/she can be detected. Secondly, if temporal data and the walking direction of the human are available, coarse localization can be performed.

In Figure 4, two ΔRSS drops are studied in detail. Figure 4a,b is a detailed view of later mentioned results. The magenta circles indicate that the ΔRSS value at that location is lower than the threshold specified in Table 1. This location is thus considered to be within the ΔRSS drop area. The magenta lines delineate the boundaries of this drop area. The size of the drop area indicates how easily someone can be detected near the LED-PD.

**Figure 6:**
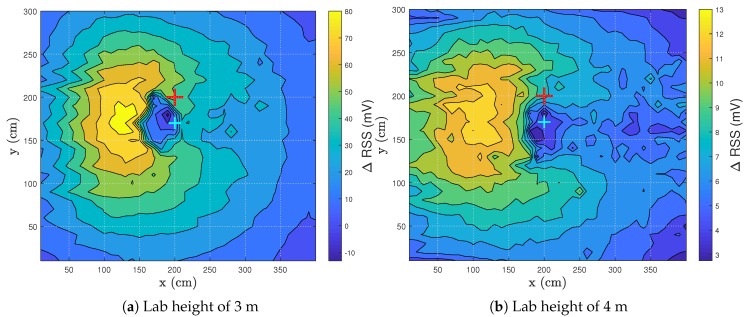
Visualization of the ΔRSS distribution for different lab heights. The red cross represents the LED, while the cyan cross denotes the PD.

**Figure 7:**
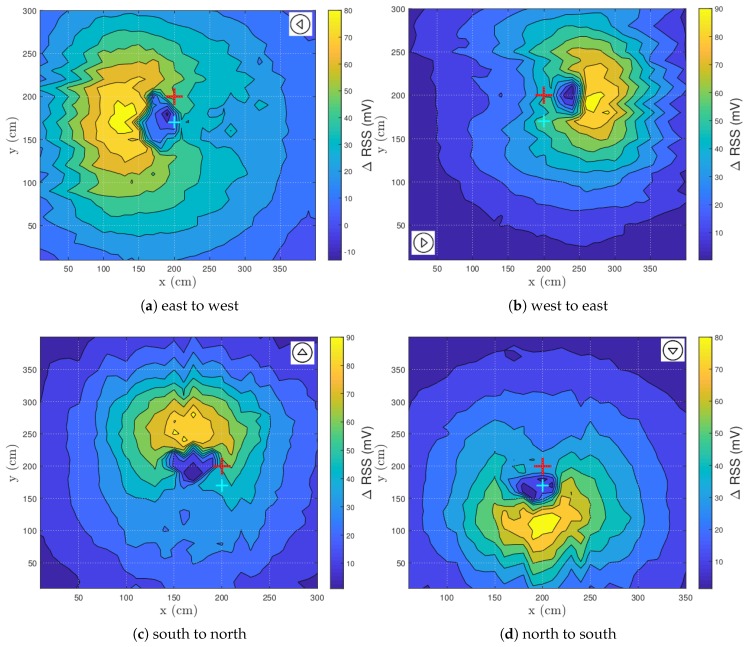
Visualization of the RSS distribution for different orientations. The red cross represents the LED, while the cyan cross denotes the PD.

Table 1 summarizes important parameters of all results that will be discussed in the following sections: minimum RSS value (mV), maximum RSS value (mV), threshold for a value to be regarded as a drop (mV) and the area of the drop (dm${}^{2}$). Figure 5a equals Figure 6a and Figure 7a so is only included once.

### 3.2. Influence Clothing Colors

Figure 5 shows the ΔRSS pattern of three different colors of worn t-shirts. A white (Figure 5a), black (Figure 5b) and red (Figure 5c) t-shirt were worn by the test subject.

When light strikes a surface, some of its energy is reflected and some is absorbed. The color a person perceives indicates the wavelengths of light being reflected. White light contains all wavelengths of the visible spectrum, so when “white” is being reflected, that means all wavelengths are being reflected and none of them absorbed, making white the most reflective color [24]. As white is the most reflective color, the RSS values are expected to be the highest when wearing a white t-shirt. If a black t-shirt is worn, the RSS values are expected to be very low. Other colors will have different RSS values and are determined by i.a. the responsivity of the used PD, the reflective coefficient of the color and the light source.

Figure 5a shows the reflection pattern when walking from right to left while wearing a white t-shirt. The location of the drop is visible in Figure 5a with a minimum RSS value of −13.1 mV at location (190,180). The maximum of 85.83 mV (see Table 1), here at location (140,180), can be elucidated by considering the reflections in the environment. The highest reflecting part of the floor is unblocked as there is a clear reflection path between the PD and the LED. In addition, the person is standing with his back at the LED-PD combination. As the back is smoother than the front of a person, the light will have a higher probability of reflecting towards the PD rather than be scattered across the room. The back will add a more specular reflection.

It can also be established that zones appear in which the ΔRSS has the same magnitude, indicated by equal colored zones. This phenomenon will be useful when VLS tracking is performed. The person will have a specific RSS lapse when walking in a VLS-enabled environment. However, the initial walking direction of the person must be known before tracking can be performed. If the RSS lapse is analyzed, the walked route can be discovered. In corridors, the walking direction is restricted to two directions, so it is beneficial to focus on corridors for visible light sensing or localization in future research. When enough data is gathered, machine learning can be utilized to analyze new data and predict the route a person of interest took.

In Figure 5b, the only zone where a human can be detected is the ΔRSS drop. Due to the limited reflection of a black t-shirt, the ΔRSS value does not alter throughout the room. Hence no differentiation can be made between noise and the reflections coming from the test subject. This result presents a challenge for VLS. Further testing will need to prove the feasibility of a VLS system when dark clothes are worn. However, in the area of the drop, a minimum ΔRSS value of −60.99 mV is obtained. This is more than 4 times the magnitude than when a white t-shirt is worn (−13.12 mV). The area of the drop increases to 7.5 dm${}^{2}$ (Table 1). According to these two values, a human is detected easier in the zone of the drop when wearing a black t-shirt.

Figure 5c shows that when the test subject wears a red t-shirt, a similar pattern is obtained as for the case when wearing a white t-shirt. A red t-shirt is chosen as a center color between the extreme colors black and white. The RSS range is half as wide (50.4%) as when a white t-shirt was worn. The minimum and maximum are respectively higher and lower, as seen in Table 1. The reflection originating from a t-shirt is clearly captured in these experiments. The remainder of the tests will be performed wearing a white t-shirt.

### 3.3. Influence of Photodiode Height

Here, the influence of the path length between the Tx and Rx is investigated. As seen in (2), the length of the path will affect the RSS values. The height of the lab was changed from 3 m to 4 m. The test subject remained oriented from right to left. The PD gain remained the same so as to compare the results. The RSS pattern of both measurements is given in Figure 6.

At a height of 3 m, a ΔRSS difference ($|RSS_{max}|+|RSS_{min}|$) of 98.95 mV was obtained. If the influence of the empty environment was not filtered out ($RSS_{empty}$ in (3)), the maximum RSS value was 262.26 mV. $RSS_{empty}$ equals 176.43 mV. The person thus added a maximum RSS value of 85.83 mV to the measurement, which is an increase of ± 50.

At the height of 4 m, the ΔRSS difference equalled 15.77 mV. Here, the maximum RSS value was 94.77 mV. $RSS_{empty}$ equalled 81.76 mV. The person thus added a maximum RSS value of 13.01 mV to the measurement, which was an increase of ± 16.

The test subject has a stronger influence on the RSS if the ceiling height is lower—the path length is shorter at 3 m than at 4 m, which entails less attenuation of power and accordingly higher RSS values. Of course, by changing the height of the ceiling, not only the path length changes but also the angles of irradiance and incidence change in the contribution of the relative elements. Although the influence changes, the RSS pattern exhibits similar behavior, which shows the repeatability of a VLS system.

As the room height increases, a maximum height will be found where human sensing is still possible achieving a certain accuracy. If the height of the room surpasses this maximum, VLS will no longer be feasible. Of course, by altering specific parameters (e.g., the gain of the receiver, the transmitted power of the light), this maximum could increase.

### 3.4. Walking Direction Relative to LED-PD

By changing the walking direction of the test subject in the lab and thus the test subject’s orientation, a rotation point might be detected. The rotation point can be defined as a point around which the RSS pattern turns if the person’s orientation changes. The standard walking direction is orientated from right to left (E → W). Figure 7 shows the RSS patterns of the various walking directions: E → W (Figure 7a), W → E (Figure 7b), S → N (Figure 7c) and N → S (Figure 7d). The arrow in the figures displays the orientation of the test subject. The PD and LED positions are constant throughout every experiment performed in this section.

First, a drop was observed for every ΔRSS pattern. Second, the ΔRSS magnitude was stable for all the results, as can been seen in Table 1. In case the standard walking direction is considered (Figure 7a), the minimum drops below zero. The area of the drop equaled 4.5 dm${}^{2}$. If the test subject turns 180 degrees, the area of the RSS drop decreases a little to 3 dm${}^{2}$. The area was smaller because the RSS points below the threshold were located closer together. However, if the test subject walks from north to south or vice versa, the area of the drop increases to respectively 7.5 dm${}^{2}$ and 12 dm${}^{2}$. The increase occurs due to the extra blockage of the light as the human is standing perpendicularly on the direct reflection path between the LED and the PD. As a consequence, the reflective surface increases (back/chest vs. side).

According to these experiments, a clear rotation point cannot be found. However, this is an expected result as a human’s shape resembles an ellipse rather than a circle and the LED-PD does not rotate as the human does. Nonetheless, a similarity across the various human orientations can be established. A drop (i.e., hair reflections) precedes a maximum (i.e., t-shirt reflections) in every walking direction in the vicinity of the LED-PD combination. If the orientation of a test subject is known, coarse localization can be performed.

**Figure 8:**
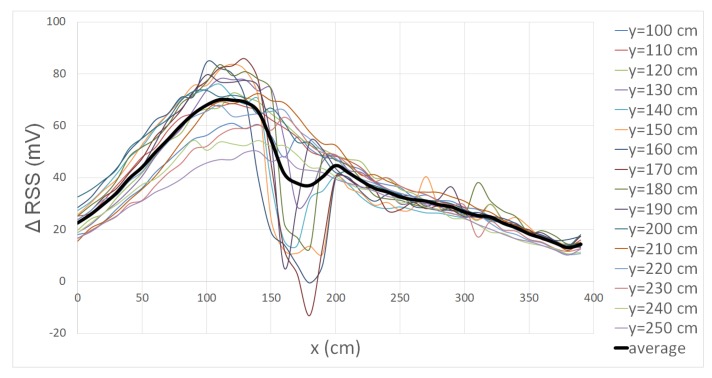
ΔRSS lapses from Figure 7a. The thick black line represents the average value.

Figure 8 shows the ΔRSS lapses from y = 100 cm to y = 250 cm from Figure 7a and the average of all those lapses, indicated by the thick black line. The walking direction of the test subject is from right to left. Based on the average, very coarse localization can be performed. If a ΔRSS is higher than 50 mV, the position will be between x = 50 cm and x = 150 cm. If the ΔRSS is under 30 mV, the location will be between x = 250 cm and x = 390 cm or between x = 0 cm and x = 25 cm. If multiple subsequent RSS values are given, the drop could be used to improve the localization as the relative position to LED-PD combination is then known. Other orientations show similar behavior, so that similar localization could be performed. The localization accuracy has not been tested, but it can be predicted that in the vicinity of the LED-PD combination, the position error will be low (less than one meter). However, if the distance to the LED-PD combination increases, the position error will increase as well. A possible extension could be to divide the ΔRSS lapses into several clusters based on the y-location so not only x-location can be performed, but y-location can be achieved as well.

### 3.5. LED-PD Separation Distance

**Figure 9:**
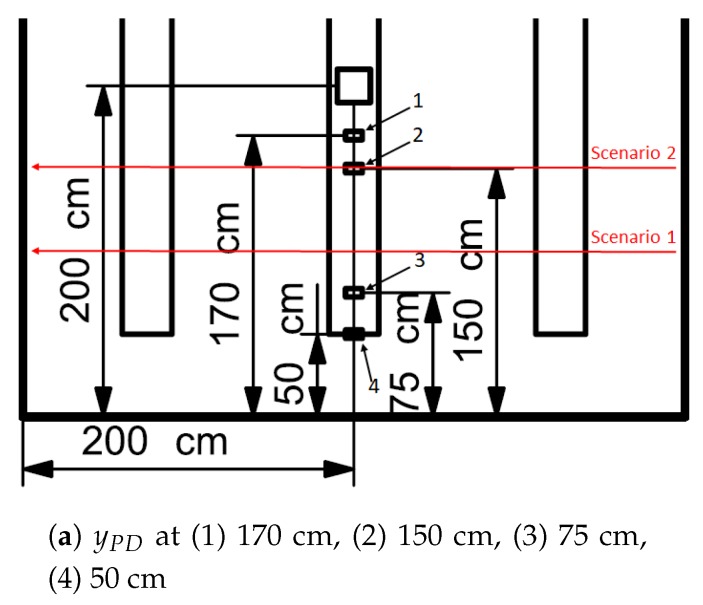

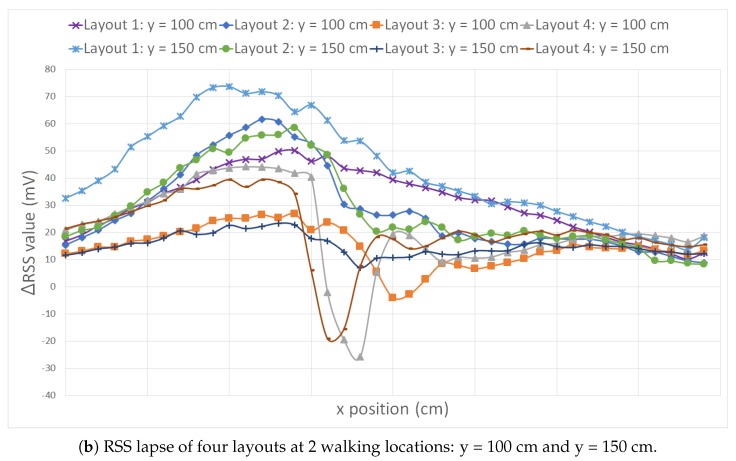
(**a**) RSS lapses of the every layout, (**b**) Visual representation of the layouts and the scenarios.

**Figure 10:**
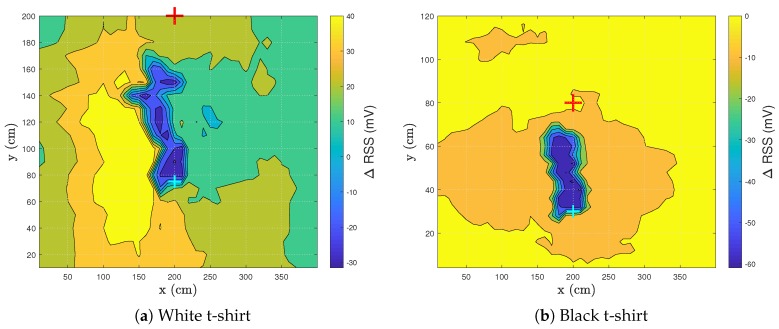
RSS distribution for layout 3. The red cross represents the LED, while the cyan cross denotes the PD. The PD is fixed at location (200,75).

The separation between the LED and PD is changed to take control of the RSS pattern. The goal is to get a more distinct pattern. The location of the PD was changed on a one-dimensional axis. The purpose of a more distinct pattern is to create a system where a person can be detected rather than located. This is done in such a way that an elongated zone is created where the RSS value is minimal. The RSS drop is expected to elongate, as a test subject will block the first order floor reflections between the LED and PD more frequently. By elongating the drop, there is a higher chance that a human could be detected if the RSS value subsides below a threshold. Four layouts are considered, out of which one is chosen. However, due to space consideration only 2 RSS lapses of all four layouts are given. A graph is included that holds the RSS lapse when the test subject walks at y = 100 cm and y = 150 cm of each of the four layouts (Figure 9b). Hereafter, the test subject walking at y = 100 cm is referenced to as scenario 1 while walking at y = 150 cm is addressed as scenario 2. The different layouts are summarized in Table 2. Layout 1 is the default layout used in previous experiments. These scenarios and layouts are indicated in Figure 10a.

The influence of the LED-PD separation is plainly visible in the graph. The relative RSS values have different minima and maxima. The maxima and minima for scenario 1 for all the layouts are summarized in Table 2. In layouts 1 and 2, there was no noticeable drop as the test subject does not block the first order reflections. In layout 3, where the test subject is now blocking first order reflections, a drop is perceived. The most substantial drop however is obtained in layout 4. The influence of the separation between the LED and PD is clearly visible based on these results. The influence of the separation between the LED and the PD becomes even more significant when the influence of the environment is not filtered out i.e., $RSS_{empty}$ not filtered out. These results are also included in Table 2. The difference between the maximum value for layouts 1 and 4 equals to 82.26 mV. This indicates the importance of the LED-PD separation and the possibility to alter this distance in order to satisfy different use cases.

Based on the previous findings, the y-location of the PD was changed from y = 1.75 m (layout 1) to y = 0.75 m (layout 3). Figure 10a shows the ΔRSS pattern of this setup. The same test subject was used and the subject wore a white t-shirt. The drop was elongated and more pronounced compared to the default layout (see Table 1). The area of the RSS drop increased from 4.5 to 13 dm${}^{2}$, which had as a positive effect that a human is more easily detectable. As the drop is now elongated, an accurate detection pass-by system can be designed and tested (Section 4). A drawback of the more accurate detection is that localization is now more difficult.

The tests were also conducted wearing a black t-shirt (Figure 10b). The zone where a human can be detected is limited to the drop. However, the drop itself followed a similar pattern as to when a white t-shirt was worn. The area of the drop was even more elongated, now spanning 16.5 dm${}^{2}$ (Table 1). A human detection system can thus be designed regardless of the color of the t-shirt as long as the test subject is walking in the occurrence area of the drop.

## 4. Human Pass-by Detection

### 4.1. Lab Environment

**Figure 11:**
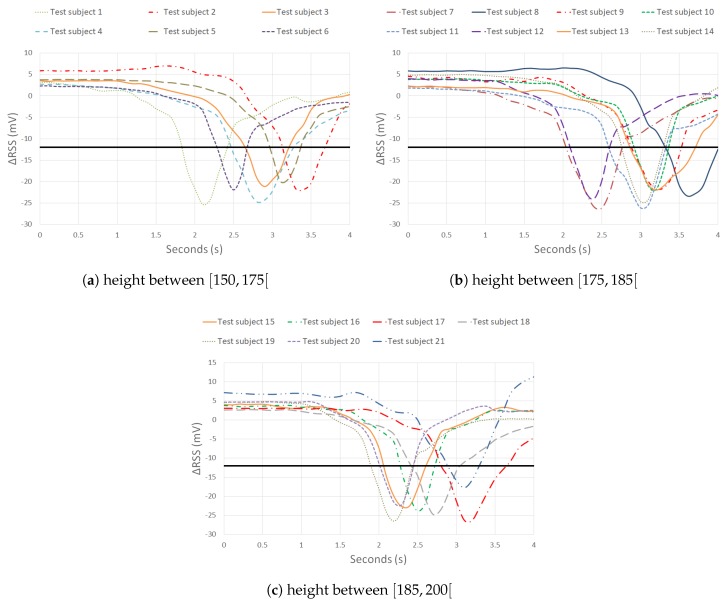
ΔRSS lapse of different people.

Based on the previous sections, a human pass-by detection system can be realized. When the layout is changed to layout 3, the drop changes into an elongated zone (Figure 10). If a human passes this zone, the aim is that the RSS value drops under a specific threshold. The threshold is defined earlier (4) and equals −11.95 mV based on Figure 10a (Table 1). The threshold is chosen based on the white t-shirt scenario of the test subject. As white is the most reflective color, every other experiment should entail a threshold that is lower than this threshold. However, each environment will require a unique threshold. A good threshold can limit the number of false negative or false positive errors i.e., not detecting a human or wrongly detecting someone. Ambient light (e.g., sunlight, position of blinds) changes throughout the day so a specific environment needs multiple threshold values. As ambient light varies slower than a typical detection time-frame, no ambient light induced error is expected. The threshold values are chosen on the spot as creating a database for every illumination situation is infeasible. Figure 11 includes the ΔRSS lapse of all 21 tests. The results have been divided into three graphs based on the height of the test subject. The first graph includes the experiments of test subjects that are smaller than 1.75 m. The second graph contains the RSS lapses of test subjects who are taller than 1.75 m but smaller than 1.85 m. The last graph contains the results of people which are taller than 1.85 m. The experiments are performed as explained in Section 2.3.2. The horizontal black line represents the threshold of −11.95 mV.

In all experiments, the samples are well below the given threshold. The system detects every test subject. Regardless of the height or the appearance of the test subject, a significant RSS drop occurs. The minimum value of each ΔRSS lapse has a magnitude in the range of −30 to −20 mV. Averaging these values, following results are obtained: −22.4 mV for Figure 11a, −23.7 mV for Figure 11b and −23.4 mV for Figure 11c if the minimum value of each ΔRSS lapse is considered. The average of each minimum ΔRSS value is in the same magnitude order so a conclusion might be drawn that the height of a test subject will not have a great influence on the RSS lapse in a dynamic environment.

It can be established that for all the 21 tests that are performed, the system detected all the test subjects. Detection accuracy of 100% is achieved. Thanks to the elongated drop and a well chosen threshold, a simple detection system is designed. However, in a real-life setting, a more robust definition of the threshold is needed to design an accurate human pass-by system. The tests were performed in a controlled lab environment and only one person passed under the system at a given time.

### 4.2. Validation in Realistic Environment—Office Corridor

**Figure 12:**
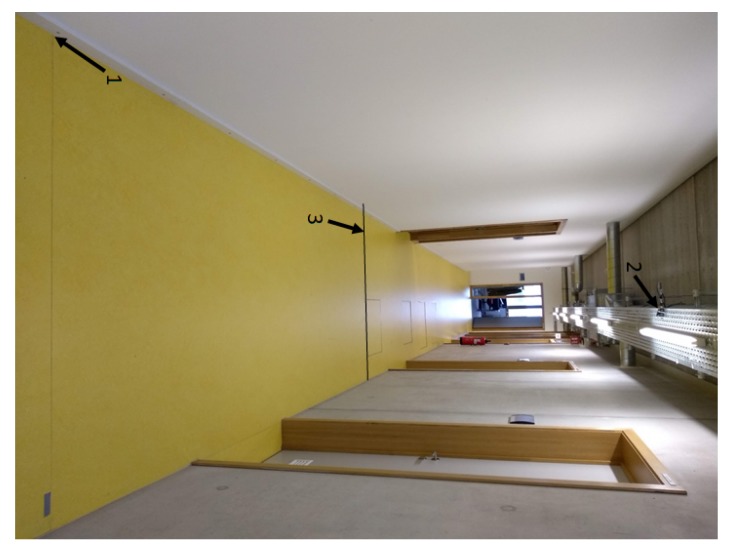
Rotated picture of the office corridor. (1) origin, (2) PD mounted next to light fixture, (3) end of 4 m area.

All of the previous results were obtained in a controlled lab environment. To illustrate that our gathered conclusions are also valid in a real-life situation, tests are conducted in a standard hall environment. The hall itself is 1.78 m wide and equipped with standard fluorescent light fixtures at a height of 2.4 m. The photodiode was placed in the same plane. A four meter long stretch is considered to validate the lab findings. The photodiode is placed in the middle of the detection zone (y = 2.03 m) and next to the fixture (x = 1.12 m) in regard to the origin (i.e., lower left corner). Figure 12 shows the corridor environment with the PD mounted next to the light fixture. The origin is also indicated.

**Figure 13:**
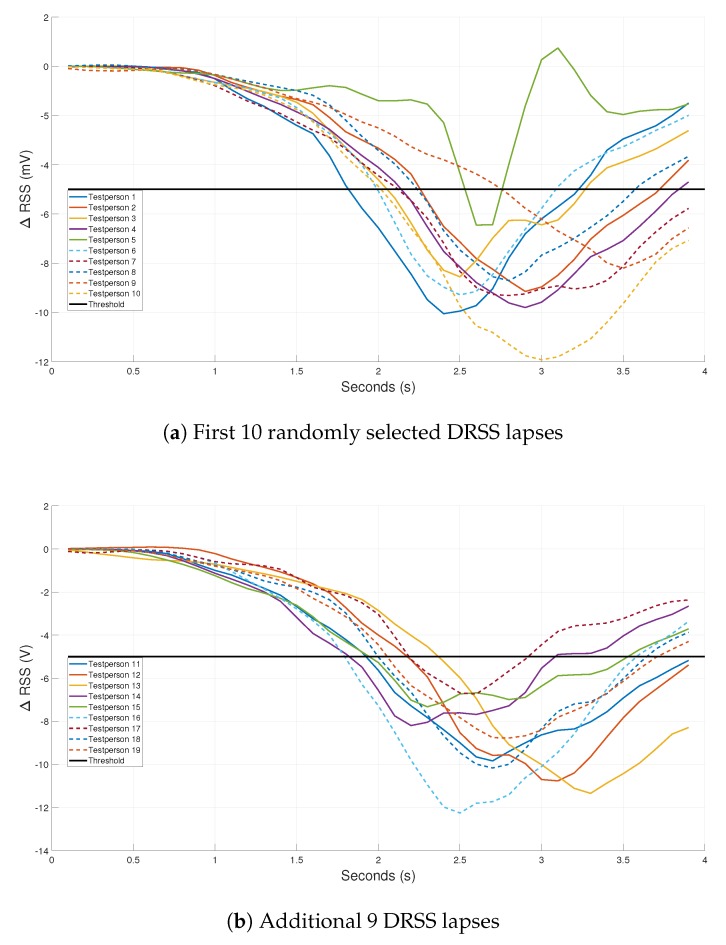
ΔRSS lapse of 19 different people in a realistic office corridor.

Again, 19 randomly selected subjects were used. The test subjects were between 1 m 61 and 1 m 97 tall. Figure 13 shows the ΔRSS lapses of the experiments and a defined threshold. There was a variation in walking speed among the test subjects, which explains the difference in the drop occurrence timing. In addition, the test subjects were free to walk along the entire width of the hallway to approach a real-life situation as close as possible. The threshold was defined by Equation (4), based on 10 randomly selected ΔRSS lapses (ΔRSS lapses included in Figure 13a). However, as the threshold is dependable on the area of the test environment and the FOV of the receiver, the threshold percentage (25%) earlier defined is no longer valid as the corridor width does not equal to 4 m. Consequently, the threshold percentage was increased to 45% to ensure that a sufficient area within the FOV of the receiver was considered. The threshold equals −49.94 mV and the system was able to detect all of the 19 test subjects. Hence, in a realistic corridor experiment, again an accuracy of 100% was obtained. However, as there is a ΔRSS drop noticeable in every ΔRSS lapse, an algorithm that focuses upon falling and rising of the ΔRSS values in the lapse might achieve similar results.

## 5. Discussion

This paper focuses on an environment without external variable light sources (e.g., sunlight) that is static, except for the passage of humans (e.g., toilet, printer room, etc.). The need for sensing is high in a multitude of environments. Self-driving cars may be the most important, as correct human sensing can save lives. Self-driving vehicles are fitted with multiple sensors such as cameras, LiDAR and ultrasound. However, it is interesting to add multiple photodiodes to a car as well as this can enable infrastructure-to-vehicle (I2V) and vehicle-to-vehicle (V2V) communication [25]. Based on the current sensing model, where a PD and light are ceiling-mounted, a similar model could be used to detect humans traversing lighting posts. Especially at crossings, such information is vital. If a human is detected, the lighting post can communicate with the vehicle (through VLC or RF) and thus warn it about human presence. If a car receives this information, it can distribute this towards other vehicles (V2V) [26].

Another environment where human sensing is vital is in industrial scenarios. In (partially) automated warehouses, pedestrian movements must comply with strict rules (e.g., where to walk and when to cross). A similar model as the one proposed in this paper can be used to detect human traffic in specified zones. Either it can be used to count the number of people crossing a defined area or it can be used to detect improper walking behavior. If a detected person is not detected by subsequent VLS nodes (and he/she did not leave the VLS enabled zone), it might indicate that this person has fallen and requires help. However, in an industrial setting, the environment is highly dynamic and challenging for VLS due to high noise (e.g., dust, tilt) and high ceiling heights. It must be investigated if a human can still be accurately detected in a standard industrial environment. Due to reflective clothing restrictions (i.e., safety vest, helmet), a higher ΔRSS value is expected when a human is traversing the LED-PD combination.

## 6. Conclusions and Future

A lab of 4 m × 4 m was equipped with a single ceiling-mounted photodiode and light to experimentally investigate the influence of a human on the reflection pattern in a controlled environment. Our results demonstrated the feasibility of a human sensing system based on visible light.

The influence of the color of a t-shirt is reported. A different colored t-shirt leads to a different RSS pattern, which can help in identifying a person. Coarse localization is possible based on the RSS lapse. It is found that the influence of the human on the RSS value decreases from ±50%to±16% when increasing the receiver height from 3 to 4 m. A maximum height can thus be found where VLS is still feasible with the current setup.

The PD-LED separation changes from 30 cm to 1.5 m to elongate the ΔRSS drop. By elongating the ΔRSS drop, a human pass-by system is easier to create, as the minimum RSS value is lower and the area of the drop is more significant. The human detection system tested in this article achieves a 100% accuracy based on 21 experiments with a LED-PD separation of 1.25 m. In a realistic corridor experiment, an accuracy of 100% is obtained based on 19 experiments. However, the consideration must be made between an accurate detection pass-by system or localization as both require a different setup.

Future work will consist of investigating more environmental parameters (e.g., a different floor, different wall and ambient light) and use this data to construct a real-life setup that can automatically adapt to a new threshold. An algorithm that focuses on the falling and rising of ΔRSS values in a ΔRSS lapse might achieve similar detection rates and is worth investigating. The influence of multiple people passing under the PD at the same time is to be investigated as well. Extending to a multiple light, multiple receivers environment to improve localization is another important research direction.

## Figures and Tables

**Table 1:** Overview of statistics for all results.

Situation	Min RSS (mV)	Max RSS (mV)	Threshold (mV)	Drop Area (dm²)
**Figure 5a, white t-shirt**	−13.12	85.83	11.62	4.5
**Figure 5b, black t-shirt**	−60.99	9.29	−43.42	7.5
**Figure 5c, red t-shirt**	−4.26	45.63	8.22	3
**Figure 6b, white t-shirt, lab height 4 m**	2.76	13.01	5.3213	9
**Figure 7b, right orientation**	0.30	99.72	25.15	3
**Figure 7c, up orientation**	1.07	94.58	24.45	12
**Figure 7d, down orientation**	1.48	86.32	22.70	7.5
**Figure 10a, white t-shirt, $y_{PD}$ = 0.75 m**	−31.58	46.93	−11.95	13
**Figure 10b, black t-shirt, $y_{PD}$ = 0.75 m**	−60.97	2.48	−45.11	16.5

**Table 2:** PD locations for the different layouts and the maxima and minima (Δ)RSS values.

Layout #	y Location PD (cm)	LED-PD Separation (cm)	Maximum ΔRSS (mV)	Minimum ΔRSS (mV)	Maximum RSS (mV)	Minimum RSS (mV)
1	170	30	50.08	10.24	226.51	186.67
2	150	50	61.68	8.78	179.93	138.63
3	75	125	26.78	−4.04	170.46	139.64
4	50	150	44.14	−25.82	144.25	74.29
